# Knowledge of the Benefits and Risks of Oral Contraceptive Use Among Women of Reproductive Age in Western Saudi Arabia: A Descriptive Cross-Sectional Study

**DOI:** 10.7759/cureus.76400

**Published:** 2024-12-26

**Authors:** Anwar Salem, Nusaybah Shafi, Shajn S Alsaadi, Fatimah Klantan, Danah S Alhajjaji, Rojinah Allihyani, Mazen Daghestani

**Affiliations:** 1 College of Medicine, Umm Al-Qura University, Makkah, SAU; 2 Obstetrics and Gynaecology, Umm Al-Qura University, Makkah, SAU

**Keywords:** associated, awareness, oral contraception, risks and benefits, western region of saudi arabia

## Abstract

Introduction: For women of reproductive age, oral contraceptives (OCs) are a well-liked and practical way to control pregnancy. OCs are also used to treat acne, irregular uterine bleeding, and premenstrual syndrome. However, there are false beliefs regarding their benefits and risks. The primary health benefits of OCs are the reduction of acne, the risk of colon, ovarian, and endometrial cancer, and the risk of osteoporosis. Other benefits include the reduction of the risk of endometriosis, fibroid tumors, menorrhagia, dysmenorrhea, benign breast illness, and ovarian cysts. The objective of this study was to assess the level of awareness among women of reproductive age in western Saudi Arabia regarding the benefits and risks of OCs.

Method: A descriptive cross-sectional study was used to gather data from women aged 15-49 in western Saudi Arabia. The study used an online questionnaire designed using Google Forms (Google LLC, Mountain View, California, United States) that was distributed electronically via social media apps in Arabic language. Data was collected in Excel sheets (Microsoft Corporation, Redmond, Washington, United States) and analyzed using IBM SPSS Statistics for Windows, Version 21.0 (Released 2012; IBM Corp., Armonk, New York, United States). After determining our study population, we calculated the sample size using OpenEpi version 3.0.

Results: A total of 588 Saudi women participated in the study and revealed that only 13.6% had an excellent understanding of OCs. The primary sources of information about oral contraceptives were friends and relatives (n=268, 45.6%), followed closely by physicians (n=255, 43.5%). Other sources included reading reliable materials, social media, and personal experience.

The sources of information were strongly linked to the participants' knowledge levels. Among those who sourced their information from reading reliable sources, 25.1% (n=42) exhibited good overall knowledge, while 22% (n=27) of those who relied on social media, 16% (n=41) of those who were informed by their physician, and only 3.2% (n=1) of those without any information source demonstrated a similar level of understanding.

Conclusions: The study highlights a lack of knowledge among reproductive-age women in western Saudi Arabia about the benefits and risks of OCs. It underscores the need for targeted educational initiatives to enhance awareness and understanding. By improving knowledge, healthcare providers can empower women to make informed decisions about their reproductive health. Ongoing research and advocacy are essential to ensure access to accurate information and support, ultimately aiming for better reproductive health outcomes in the region.

## Introduction

Oral contraceptives (OCs) are used worldwide to prevent pregnancy in women of reproductive age and to treat premenstrual syndrome, abnormal uterine bleeding, and severe acne [1]. In a study conducted in Riyadh, Saudi Arabia, which included 432 women, 249 (57.6%) were contraceptive users, with the most common method being OCs (55.6%) [2]. OCs are one of the most popular and most effective reversible forms of birth control [3]. Combined estrogen-progesterone pills and progestin-only pills are two types of OCs and are made of synthetic ovarian hormones [4]. Rapid changes in the sociodemographic pattern of the Saudi Arabian population, particularly those affecting women’s employment and education, are playing a significant role in altering fertility beliefs, increasing the tendency to space out births, and thus in the use of contraceptives [5]. However, despite the widespread use of OCs, there are many misconceptions about both the benefits and risks.

Many studies describe the benefits of OCs. In addition to preventing pregnancy, regulating menstruation, and reducing the risk of endometrial, ovarian, and colon cancer, they can be used to treat dysmenorrhea, endometriosis, acne, menorrhagia, hyperandrogenism, polycystic ovary syndrome, functional ovarian cysts, premenstrual syndrome, myomas, and pelvic inflammatory disease. OCs are also beneficial in treating rheumatoid arthritis, multiple sclerosis, and certain conditions experienced in perimenopause [6-8]. Possible side effects of using hormonal contraceptives include depression, breast cancer, and breakthrough bleeding [9-11].

There have been numerous studies on OCs in Saudi Arabia to evaluate women’s attitudes and practices regarding their use. However, there are limited studies dedicated to assessing Saudi women’s knowledge of the benefits and risks associated with OCs. Therefore, the aim of this study was to evaluate the knowledge of women of reproductive age living in western Saudi Arabia regarding the benefits and risks of using OCs.

## Materials and methods

This was a cross-sectional study carried out at the Umm Al-Qura University College of Medicine, Makkah, Saudi Arabia, from November 2023 to January 2024. The study was approved by the Institutional Research Board at Umm Al-Qura University (approval number: HAPO-02-K-012-2023-10-1793). The target population for this study consisted of women residing in the western region of Saudi Arabia aged 15-49 years who were residents of the western region during the study period.

After determining our study population, we calculated the sample size using OpenEpi version 3.0 (https://www.openepi.com). The study successfully included 588 women who met the aforementioned criteria. All participants voluntarily agreed to take part in the study, ensuring that the data collected reflected genuine insights from individuals who were willing to contribute.

Study tool and data collection

An online self-administered questionnaire, designed in Arabic to accommodate the linguistic preferences of the target population, was utilized. The questionnaire was validated and adapted from a published study [12] with some questions rephrased to align with the cultural context of our environment. The English translation is given in the Appendices. Moreover, a pre-test was conducted by distributing the survey to 20 participants, and then the survey was distributed through various social media applications, ensuring broad accessibility and reach to eligible participants. The use of social media platforms for distribution allowed for efficient and wide-reaching engagement with potential participants who fit these criteria.

The structured questionnaire comprised four distinct sections to capture a comprehensive range of data: (i) Demographic information: This section collected essential background data about the participants, including age, educational background, and other relevant demographic variables; (ii) Level of knowledge concerning the benefits and side effects of oral contraceptives: Here, participants were assessed on their understanding of the advantages and potential adverse effects associated with the use of oral contraceptives; (iii) Background information concerning the use of oral contraceptives: This section gathered information about the participants' personal experiences and history with oral contraceptives, including duration of use and reasons for choosing or not choosing these methods; (iv) Usage patterns and preferences: This final segment aimed to elucidate patterns of OC use, including frequency, types of OCs used, and personal preferences or trends.

This comprehensive approach aimed to provide a detailed understanding of contraceptive knowledge and practices within the specified demographic, offering valuable insights for public health initiatives and educational programs.

Data analysis

The data were collected, reviewed, and then analyzed using the IBM SPSS Statistics for Windows, Version 21.0 (Released 2012; IBM Corp., Armonk, New York, United States). All statistical methods used were two-tailed with an alpha level of 0.05. The p value was considered significant if it was less than .05. Regarding knowledge and awareness, each correct answer was given a score of 1. The overall level of awareness about the benefits and risks of OCs was determined by summing up the scores for correct answers. Knowledge was categorized as poor if a participant’s score was less than 60% of the overall score and as good if a participant’s score was 60% or more of the overall score. Descriptive analysis was conducted using frequency distribution and percentage for the study variables, including the participants’ personal data, education, and use of OCs. In addition, scores regarding knowledge and awareness of the benefits and risks of OCs were tabulated, and overall knowledge level and source of information were detailed in graphs. Cross-tabulation to show the distribution of the participants’ level of overall knowledge based on their data was carried out using a Pearson chi-square test for significance and an exact probability test if there were small frequency distributions.

## Results

A total of 588 women participated in the survey, with an average age of 33.1 years. The majority were married (65.1%) and had university-level education (70.6%). About 48.8% had used or were using OCs. Key findings included that only 13.6% of the participants had good overall knowledge about OCs, and the most recognized benefits were contraception and menstrual regulation, while risks such as weight gain, nausea, and increased risk of blood clots were the most commonly known. Table 1 presents a summary of the participants’ demographic information, including age, marital status, educational level, and their current or past use of OCs.

**Table 1:** Personal characteristics of the participants (N = 588)

Personal data	Frequency	Percentage
Age group in years		
15–19	13	2.2%
20–29	219	37.2%
30–39	124	21.1%
40–49	232	39.5%
Are you married?		
Yes	383	65.1%
No	205	34.9%
Educational level		
Below university	121	20.6%
University graduate	415	70.6%
Above university	52	8.8%
Do you use/have you ever used oral contraceptives?		
Yes	287	48.8%
No	301	51.2%

Table 2 summarizes the participants’ knowledge regarding both the risks and benefits of OC use. While most were aware of the risk of weight gain, other potential side effects and benefits were less recognized, indicating gaps in knowledge.

**Table 2:** Participants’ knowledge of the benefits and risks associated with oral contraceptives (N=588)

Knowledge items	Frequency	Percentage
The expected side effects of oral contraceptives		
Increased weight	443	75.3%
Increased risk of ectopic pregnancy	114	19.4%
Increased risk of ovarian cancer	129	21.9%
Increased risk of cervical cancer	121	20.6%
Increased risk of breast cancer	100	17.0%
Nausea	305	51.9%
Elevated blood pressure	242	41.2%
Increased risk of pelvic inflammatory disease	60	10.2%
The expected benefits of oral contraceptives		
Contraception	541	92.0%
Reduced risk of ovarian cancer	37	6.3%
Regulating the menstrual cycle	369	62.8%
Reduced acne	79	13.4%
Reduced anemia	46	7.8%
Reduced risk of cervical cancer	49	8.3%
Reduced blood pressure	12	2.0%
Reduced depression	24	4.1%
Reduced risk of breast cancer	23	3.9%
Smoking for those taking oral contraceptives may lead to		
Increased risk of blood clots	384	65.3%
Elevated blood pressure	237	40.3%
Increased risk of stroke	141	24.0%
Increased menstrual blood density	120	20.4%
Increased risk of brain cancer	52	8.8%
Increased risk of uterine cancer	111	18.9%
There is no risk associated with smoking while taking oral contraceptives	100	17.0%

**Figure 1:**
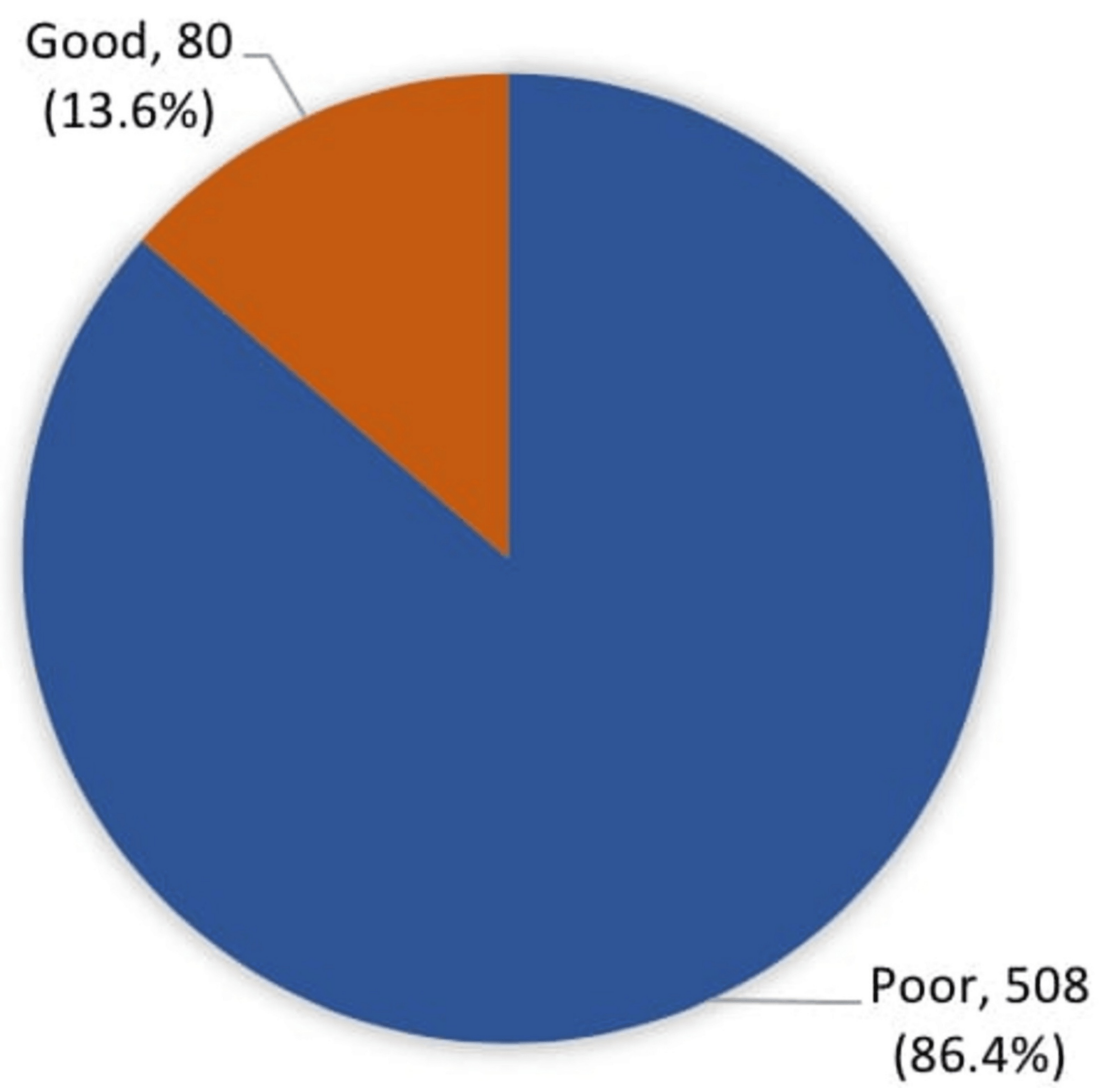
Participants’ overall knowledge about the benefits of oral contraceptives Data presented as n (%)

Figure 1 illustrates the distribution of participants based on their overall knowledge of OCs. Only 13.6% had good knowledge, while the remaining 86.4% had poor knowledge, emphasizing the need for better education on this topic.

**Figure 2:**
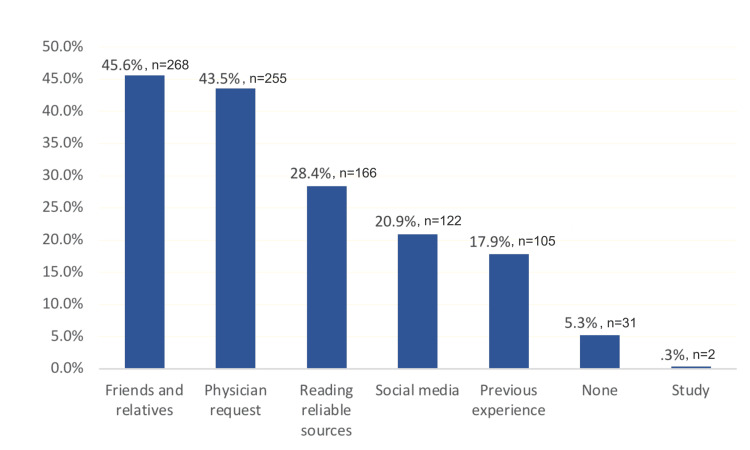
Participants’ sources of information about oral contraceptives

Figure 2 displays the different sources from which participants gained information about OCs, with the majority relying on friends and relatives, followed by physicians. Reliable sources and social media were less frequently used.

Table 3 explores various demographic and personal factors that are linked to participants’ overall knowledge of OCs. The table divides the participants into two categories: those with “Good” knowledge and those with “Poor” knowledge. It analyzes how different factors, such as age, marital status, educational level, and the use of OCs, influence the level of knowledge.

**Table 3:** Factors associated with the participants’ overall knowledge about oral contraceptives P: Pearson X2 test; ^: exact probability test; * P < 0.05 (significant)

Factors	Overall knowledge level				P value
	Poor (n=508)		Good (n=80)		
	Frequency	Percentage	Frequency	Percentage	
Age in years					.476
15–19	10	76.9%	3	23.1%	
20–29	185	84.5%	34	15.5%	
30–39	108	87.1%	16	12.9%	
40–49	205	88.4%	27	11.6%	
Are you married?					.433
Yes	334	87.2%	49	12.8%	
No	174	84.9%	31	15.1%	
Educational level					.735
Below university	107	88.4%	14	11.6%	
University graduate	357	86.0%	58	14.0%	
Above university	44	84.6%	8	15.4%	
Do you use/have you ever used oral contraceptives?					.330
Yes	252	87.8%	35	12.2%	
No	256	85.0%	45	15.0%	
Sources of information about oral contraceptives					.001*^
None	30	96.8%	1	3.2%	
Physician request	215	84.0%	41	16.0%	
Friends and relatives	227	84.7%	41	15.3%	
Previous experience	87	82.9%	18	17.1%	
Reading reliable sources	125	74.9%	42	25.1%	
Social media	96	78.0%	27	22.0%	
Study	2	100.0%	0	0.0%	

The results show no statistically significant relationship between age, marital status, education, or OC use and overall knowledge levels. However, sources of information show a strong statistical significance (p = 0.001), indicating that those who received their information from reliable sources were more likely to have good knowledge. Specifically, 25.1% of participants who consulted reliable sources had good knowledge, compared to only 3.2% of those who had no source of information. Other notable sources, like social media and physician consultations, also influenced knowledge levels but to a lesser extent.

## Discussion

OCs are among the most widely used forms of reversible contraception globally, offering both contraceptive and non-contraceptive health benefits. A variety of contraceptive options exist, including combined oral contraceptives (COCs), progestin-only pills (POPs), long-acting reversible contraceptives (LARCs), and barrier methods. Despite this range of options, many women remain unaware of the full spectrum of health benefits each method provides and lack knowledge about which option might best suit their needs [13]. According to a study done in the central region of Saudi Arabia, 66.1% of women know little or have incorrect information about OCs [12]. This is in contrast to a study carried out in India, which indicated women have high knowledge (99.2%) and a positive attitude (63.7%) about OCs [14].

The findings of this study revealed that only 13.6% of participants had a good overall knowledge of OCs. This mirrors the results of previous research, indicating a common trend of limited awareness about OCs beyond their contraceptive use [12]. Notably, while the majority (92%) recognized pregnancy prevention as a primary benefit, awareness of other benefits, like menstrual cycle regulation (62.8%) or acne reduction (13.4%), was significantly lower. In terms of the least expected benefits, 4.1% mentioned a reduction in the rate of depression, 3.9% mentioned a decreased risk of breast cancer, and 2.0% mentioned a reduction in blood pressure. A study by Kaunitz has addressed the gap between the perceived and actual benefits of oral contraceptives (OCs) [15]. It emphasizes that while oral contraceptives are widely recognized for their contraceptive purposes, their non-contraceptive health benefits, such as the treatment of acne, regulation of the menstrual cycle, reduction of menstrual disorders, and lowering the risk of certain cancers, are often underappreciated or overlooked by both patients and healthcare providers. The study highlights the need for better education and awareness around these non-contraceptive benefits to align perception with reality [15]. This aligns with our findings, where many participants were unaware of or undervalued the non-contraceptive benefits of OCs.

Additionally, awareness of side effects was mixed. Most participants were familiar with common side effects like weight gain (75.3%) and nausea (51.9%), but fewer knew about more serious risks such as the potential for ectopic pregnancy or an increased risk of ovarian cancer. The mixed awareness of oral contraceptive side effects observed in this study mirrors findings from other regions. Bryden and Fletcher reported that while university-aged women were familiar with common side effects like weight gain, knowledge of serious risks was lacking [16]. Similarly, Bardaweel et al. found that although most women in Jordan were aware of common issues like hormonal disturbances, knowledge of serious risks, such as cancer, was limited [17]. This consistency across different populations highlights the universal need for comprehensive education on both common and serious side effects, suggesting that more tailored educational strategies could address these gaps effectively.

Moreover, low-dose COCs offer several benefits, including a reduced incidence of adverse effects, compared to higher doses. These benefits may include a lower risk of thromboembolic events, improved tolerability, and fewer side effects such as weight gain and mood changes. Research indicates that low-dose COCs are effective for contraception while minimizing complications. Studies comparing low-dose and high-dose COCs highlight the safety and effectiveness of lower doses, making them an attractive option for many patients [18].

In the current study, the participants were also asked about how smoking might affect women who take OCs. A large percentage of the participants, 65.3%, stated that it may lead to an increased risk of blood clots, 40.3% stated that it might lead to high blood pressure, and 24.0% stated that it might lead to an increased risk of stroke, increased menstrual blood density, and an increased risk of brain and uterine cancer. Surprisingly, 17% of the participants stated that smoking has no effect on women who take OCs. These results are similar to the results of a study conducted in Canada, with the percentage of participants who stated smoking has no effect on women who take OCs being 4% [16].

In contrast to earlier studies, such as one conducted in Riyadh, which found a positive correlation between education level and knowledge of OCs [19], the current study did not find a significant relationship between educational attainment and overall knowledge of OCs. Instead, the key factor influencing knowledge levels in our study was the source of information. Women who obtained information from reliable sources such as healthcare providers or academic materials had significantly better knowledge than those relying on informal sources like friends, relatives, or social media. These findings differ from the 2012 Riyadh study [19], which showed that knowledge about OCs was positively correlated with longer usage and higher education levels.

Similarly, an Australian study from the same year also found that higher educational attainment was associated with a greater understanding of OCs [20]. This discrepancy with the current study can be attributed to the reliance on information sources in the present study. Cultural and regional factors may contribute to this; the western Saudi Arabian region may have limited access to integrated health education programs seen in other regions. Additionally, the rise of social media as a dominant source of health information has introduced a mix of accurate and misleading content, influencing awareness even among educated individuals. These findings underscore the need for targeted public health campaigns and education efforts to address these gaps effectively.

The sources of information our study participants relied on most to gain knowledge were friends and relatives, followed by physicians, reading reliable sources, social media, previous experience, and studying. This is in line with another Saudi Arabian survey wherein 33% of participants indicated their family was their primary source of information about OCs [5].

In summary, this study highlights critical gaps in knowledge about OCs among women in Western Saudi Arabia, particularly regarding non-contraceptive benefits and serious side effects. These findings underscore the urgent need for targeted educational interventions by healthcare providers and public health campaigns to improve awareness. Clinicians should prioritize offering comprehensive education during consultations, focusing on both the risks and benefits of OCs. Concurrently, public health campaigns must address misinformation and emphasize non-contraceptive benefits, especially for populations that rely heavily on informal information sources. By providing accurate information through credible channels, these efforts could bridge the knowledge gap and enable more informed decision-making about OC use.

The implications of these findings extend beyond addressing immediate knowledge deficits. From a clinical perspective, healthcare providers must adopt a more proactive role, emphasizing the importance of engaging patients with tailored educational efforts. This study challenges the conventional assumption that higher educational attainment directly correlates with better health knowledge, highlighting instead the critical role of reliable information sources. By shifting focus to the accessibility and accuracy of health information, clinicians can reshape educational practices in ways that align with patients’ needs and cultural contexts. On a policy level, the findings advocate for integrating targeted reproductive health education into public health initiatives. Policymakers could develop culturally tailored awareness campaigns utilizing accessible platforms, such as digital tools and community outreach, to reduce reliance on informal and often unreliable sources. This approach ensures that accurate and trustworthy information reaches diverse demographics, fostering better health literacy.

For future research, the study calls for investigations into the long-term effectiveness of diverse educational strategies, including school-based programs, mobile health applications, and community workshops. Research should also explore methods to enhance the reach of credible information, addressing disparities in health knowledge across different regions and demographic groups. Ultimately, these findings hold the potential to shift current understanding and practice by emphasizing the pivotal role of reliable information sources over educational attainment. By aligning clinical, policy, and research efforts with the insights from this study, healthcare systems can foster informed contraceptive use, leading to improved reproductive health outcomes for women in the region.

Our study had some limitations, including the reliance on self-reported data and the restriction of the sample to women with internet access. Future studies could expand the scope by including in-person interviews to capture a more representative sample of women across Saudi Arabia. However, sharing the questionnaire through various social media platforms significantly expedited the data collection process, enabling the collection of a substantial number of responses in a shorter period. This approach also broadened the reach of the survey, attracting a diverse demographic, which enriched the quality of the data collected.

## Conclusions

This study emphasized the critical gaps in knowledge among women of reproductive age in western Saudi Arabia regarding the benefits and risks associated with OC use. Most participants lacked awareness of the broader implications of OC use. While contraception and menstrual cycle regulation were well-recognized benefits, other health advantages such as reduction of the risk of certain cancers were significantly underappreciated. Similarly, common side effects like weight gain and nausea were better understood compared to more serious risks, such as an increased likelihood of blood clots or certain types of cancer. A significant association was seen between the sources of information and participants’ knowledge levels. Reliance on informal channels like friends, relatives, or social media resulted in misinformation or inadequate understanding. Additionally, the study identified a notable gap in understanding the impact of behaviors such as smoking while using OCs, with misconceptions potentially affecting health outcomes.

To address these gaps, targeted educational initiatives are essential to empower women with accurate, reliable, and comprehensive information, enabling them to make informed decisions about their reproductive health. By bridging the knowledge gap, healthcare providers can promote safer and more effective contraceptive use, ultimately improving reproductive health outcomes in the region. Continued research and advocacy remain crucial to refining public health strategies and ensuring widespread access to trustworthy resources and support systems

## Appendices

Questionnaire (Translated to English from the original Arabic)

Age?

·       15-19 years old

·       20-29 years old

·       30-39 years old

·       40-49 years old

Are you from the western region of Saudi Arabia?

·       Yes

·       No

Are you married?

·       Yes

·       No

Educational status?

·       Below bachelor’s degree (middle school, high school etc.)

·       In university (bachelor’s degree)

·       Above bachelor’s degree (doctorate, masters etc.)

Are you using oral contraceptives or have used them before?

·       Yes

·       No

Where did you get your information on oral contraceptives? (Please choose all the correct answers)

·       Used them before (past experience)

·       Asking a doctor

·       Reading about them from trusted resources

·       Social media

·       Friends and family (community)

·       Other (please mention)

Which are possible side effects of the pill? (Please circle all that apply)

·    Increased blood pressure

·    Increased risk of ovarian cancer

·    Increased risk of ectopic pregnancy

·    Increased pelvic inflammatory disease

·    Increased risk of cervical cancer

·    Increased risk of breast cancer

·    Nausea

·    Weight gain

Which of the following are possible health benefits of the pill?

·    Decreased anemia

·    Decreased risk of cervical cancer

·    Decreased risk of breast cancer

·    Decreased blood pressure

·    Decreased risk of ovarian cysts

·    Decreased depression

·    Clears up acne

·    Regulates menstrual cycle

·    Not getting pregnant

Smoking while on the pill can lead to ?

·    Increased risk of blood clots

·    Increased menstrual bleeding

·    Increased blood pressure

·    Increased risk of stroke

·    Increased risk of brain cancer

·    Increased risk of cancer of the uterus

·    No dangers

·    Other risks not listed (Please mention them)
